# Design and Adoption of Low-Cost Point-of-Care Diagnostic Devices: Syrian Case

**DOI:** 10.3390/mi12080882

**Published:** 2021-07-27

**Authors:** M. Munzer Alseed, Hamzah Syed, Mehmet Cengiz Onbasli, Ali K. Yetisen, Savas Tasoglu

**Affiliations:** 1 Institute of Biomedical Engineering, Boğaziçi University, Çengelköy, Istanbul 34684, Turkey; munzer.alseed@boun.edu.tr; 2 School of Medicine, Koç University, Sariyer, Istanbul 34450, Turkey; hsyed@ku.edu.tr; 3 Koç University Research Center for Translational Medicine, Koç University, Sariyer, Istanbul 34450, Turkey; monbasli@ku.edu.tr; 4 Department of Electrical and Electronics Engineering, Koç University, Sariyer, Istanbul 34450, Turkey; 5 Department of Chemical Engineering, Imperial College London, London SW7 2AZ, UK; a.yetisen@imperial.ac.uk; 6 Center for Life Sciences and Technologies, Bogazici University, Bebek, Istanbul 34342, Turkey; 7 Koç University Arçelik Research Center for Creative Industries (KUAR), Koç University, Sariyer, Istanbul 34450, Turkey; 8 Department of Mechanical Engineering, Koç University, Sariyer, Istanbul 34450, Turkey

**Keywords:** point-of-care devices, Syrian war, Syrian refugees, adoption, diagnostics

## Abstract

Civil wars produce immense humanitarian crises, causing millions of individuals to seek refuge in other countries. The rate of disease prevalence has inclined among the refugees, increasing the cost of healthcare. Complex medical conditions and high numbers of patients at healthcare centers overwhelm the healthcare system and delay diagnosis and treatment. Point-of-care (PoC) testing can provide efficient solutions to high equipment cost, late diagnosis, and low accessibility of healthcare services. However, the development of PoC devices in developing countries is challenged by several barriers. Such PoC devices may not be adopted due to prejudices about new technologies and the need for special training to use some of these devices. Here, we investigated the concerns of end users regarding PoC devices by surveying healthcare workers and doctors. The tendency to adopt PoC device changes is based on demographic factors such as work sector, education, and technology experience. The most apparent concern about PoC devices was issues regarding low accuracy, according to the surveyed clinicians.

## 1. Introduction

The Syrian war has forced 5.5 million Syrian people, which makes about a quarter of the population, to seek asylum in 127 countries [1]. A total of 3.6 million Syrian refugees have been registered in Turkey since 2011 [1]. As a response to such an unusual influx of population to the country, the Turkish government provided temporary protection for Syrian refugees, offering identity cards that grant them free healthcare services, and building 180 Migrant Health Centers in cooperation with the EU commission in 29 different Turkish cities [2]. However, delivering high-quality healthcare is a challenge due to equipment shortages, limited healthcare workers, and overcrowding of refugees at health centers. These issues may prevent proper and full access to relevant portals [3]. Other problems include instability that accompanies resettlement, where chronic diseases need to be continuously monitored, and healthcare providers cannot follow up with all conditions, leading to late diagnosis and treatment and severe complications. Moreover, the large numbers of refugees have cost Turkey more than USD 10 billion in healthcare services alone [4].

The average annual healthcare expenditure per person in OECD (the Organisation for Economic Co-operation and Development) countries, including Turkey, is about USD 4000 [5]. In general, the cost of health services is increasing constantly all over the world. For example, healthcare expenditures in the U.S. have increased by 13.4% between 2016 and 2019 [6]. Additionally, healthcare has out-of-pocket expenditures, since the levels of healthcare insurance systems vary among countries, resulting in wide ranges of costs even within the same community due to the difference in health conditions [7]. Poor accessibility contributes even more to the challenges facing healthcare services in developing countries. Patients who live in areas that do not have laboratories must travel to centralized facilities and wait for days for their laboratory test results [8].

To alleviate the tradeoff between high cost and poor accessibility, point-of-care (PoC) devices can be introduced as a robust solution [9]. PoC testing is a diagnostic approach that is performed at the time and place of patient care with often limited peripheral equipment [10]. Various PoC devices have been developed to diagnose many diseases or monitor certain conditions and health-related metrics. They can be generally divided into two main categories, the first being small benchtop analyzers, which are smaller, automated versions of their laboratory counterparts. The second type is handheld, single-use devices, for which microfabrication and disposable material engineering is often used for their development [11,12,13]. Some examples of PoC tests are blood glucose tests, pregnancy assays, urine strips, sexually transmitted disease (STD) testing, and haemoglobin diagnosis [14]. PoC devices are mainly used for their low cost, as they require minimized biochemical reagents [15,16,17]. They are also time efficient compared to laboratory tests that may take hours, while PoC tests can provide results within minutes. PoC devices are easy to use, eliminating the need for special training to perform the tests and enabling healthcare workers to operate the devices. Those advantages can be of a special importance for refugees in vulnerable healthcare systems [18].

Although PoC devices can be beneficial, their development is hindered by factors, especially the rapid advancement and introduction of new technologies, making it hard for end users to adopt them. These users are usually located in rural areas with minimal chances of getting any training for the new device. Therefore, PoC devices must require as little training as possible, have a low production cost [13], and the ability to withstand different conditions (as temperature and humidity can be delimiting). This is important for eliminating the need for costly special storage options [13,14,15,16,17,18,19]. On the other hand, different challenges can emerge from social barriers rather than technical ones. New technologies, such as PoC devices, need to prove their value to the end users in order to be used. The technology provider must build trust with the users and show the value the device adds. The social and cultural backgrounds of users also play an important role in their acceptance of new technologies. Therefore, it is necessary to study such factors to reveal any related reasons that prevent users from adopting new products [20].

The technical challenges can be met by developing new technologies, while the social challenges cannot be met in such a way. Instead, they need direct contact with the users, to get their feedback and investigate the reasons that may or may not convince them to adopt the new technologies, as every community has its own unique characteristics. In regards to Syrian refugees, most of the refugees are either concentrated in the southern provinces of Turkey or in the north of Syria. Even though the two regions are adjacent, the difference between healthcare services can change the order of priority when it comes to the desired features of a PoC device. Those differences can be due to the varying levels of education and experience of the healthcare workers. Furthermore, the availability and accessibility of these technologies in the healthcare centers are a contributing factor. Doctors and healthcare workers who apply PoC devices should be interviewed and surveyed to learn about their most preferred features of the device. This includes costing, ease of use, speed of results, or another suggested feature. They can also inform about any disadvantages they find in the device so that they can be avoided during development. This article aims to inform healthcare professionals, product developers and the public about PoC devices by surveying the relevant parties including healthcare workers and doctors working in developing countries.

## 2. Syrian Case with Numbers: Immigration-Related Healthcare Burden

Refugees are more likely to suffer from diseases, especially when they live in different environments than their original one. In a survey that was conducted in Syrian refugee camps in Turkey to understand the prevalence of risk factors of non-communicable diseases among refugees. Results showed high risk rates of various diseases such as hypertension and cardiovascular disease [21]. Studies on infectious diseases have shown an increase in specific diseases in Syrian refugee camps, although it varies from country to country. Such studies have reported a significant increase in Tuberculosis (TB) and Hepatitis infections in refugee camps in Lebanon and Jordan [22,23], and a similar increase in cutaneous leishmaniosis skin infections in refugee camps in Turkey, Syria, Lebanon, and Jordan [23]. To provide proper healthcare services for refugees, host countries have taken different approaches [21,24]. In the case of Turkey, Syrian refugees who live inside or outside of camps benefit from the healthcare system facilities through the “Law on Foreigners and International Protection” [24,25]. In the camps or rural areas of Turkey that have limited medical equipment, patients are generally redirected to comprehensive hospitals in the city centers. The use of low-cost, reliable, point-of-care rapid diagnostic tests, especially in environments such as family health centers and refugee camps, can be useful to eliminate the risk of late diagnosis.

In the report called “Health Status Survey of Syrian Refugees in Turkey”, the assessment of situations associated with non-communicable diseases (NCDs) and health status between the Syrian refugees currently living in various camps in Turkish cities was implemented by the World Health Organization (WHO, Geneva, Switzerland). They have shown and assessed the prevalence of chronic risk factors linked with an individuals demographical profile (age, education, employment, revenue) by investigating the frequency of chronic disease risk factors for 5760 Syrian refugees in Turkey. The evaluated elements as risk factors were daily smoking, nutrition (fruit and vegetable consumption), physical activity, obesity, and high blood pressure. Furthermore, they recorded physical measurements for blood pressure, height, and weight, as well as a questionnaire. The results demonstrated that about 60% of Syrian refugees aged 18–69 years old had a high risk for NCDs, including hypertension and obesity. Men (81.7%) and women (87.1%) in the 45–69 age group were exposed to a high combined risk (contains more than three risk factors), while hypertension prevalence is 27.2% in men, 23.8% in women, and 25.6% in both genders who had high blood pressure arterioles in their measurements or who were currently using drugs due to high blood pressure. The evaluation of the weight measurements and questionnaire results illustrated that 29.0% of the female refugee population was overweight and 36.2% of females showed signs of obesity. When comparing these results to men, women were significantly more likely to be overweight or obese (56.2% vs. 60.3%). On the other hand, the prevalence of being overweight increased significantly with age, as it was 41.0% in the 18–29 age group, and 83.3% in the 18–69 age group, regardless of gender (Table 1). This report provided information on the use of healthcare services as well as the prevalence of risk factors for NCDs in the region where refugees live [21].

Even though access to healthcare services was provided for the refugees by Turkey through free medical examination, treatment, and vaccination, different barriers limited the optimal benefits of such facilities, and caused more prevalence of difference infectious diseases. Language represents an important barrier, which leads the refugees to avoid receiving the provided services. Cases such as respiratory tract infections and diarrhea showed a significant increase among refugees living in temporary shelters between 2012 and 2016. More serious and threatening diseases, such as tuberculosis, have also increased in that period, where seven cases were diagnosed in 2012, becoming 28 cases in 2016. Moreover, the influx of refugees correlated with an increase in measles and leishmaniasis in Turkey [26].

## 3. Methods

All surveys were performed anonymously via https://globalchallenges.ku.edu.tr/survey-for-doctors/ (20–31 January 2021). The healthcare worker demographic information was analyzed with their responses given to the survey questions using Likert and box plots. Six of the survey questions were summarized together to create a binary outcome variable which assessed whether the worker was more positive or negative towards using PoC devices. For each worker, the result of the binary variable was found by calculating a score based on the level of agreement or disagreement with 6 statement questions. These questions investigated 4 parameters related to PoC devices, including importance, ease of use, accuracy, and accessibility. As the average score would be equal to 18, this value was set to be a threshold to decide whether the worker is positive or negative towards PoC devices.

The Likert plots allowed for visualizing categorical variables together based on question responses. To summarize continuous variables such as years of experience of each worker we visualized the data using box plots. Box plots allowed for comparing the demographic variable categories across mean values of the continuous variable.

## 4. Results and Discussion

**Figure 1:**
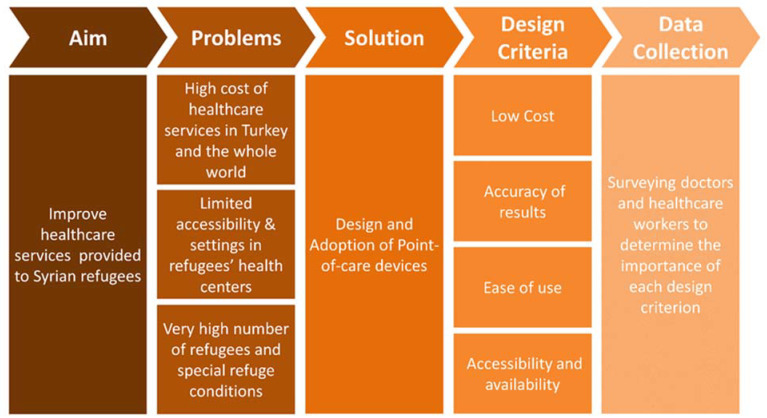
Overview of the workflow of the study.

This survey was aimed for doctors and clinicians who had direct contact with patients and worked with them daily. The goal of this survey was to investigate the most crucial factors for those end users that contribute to their acceptance to the use of PoC devices for diagnosis (Figure 1). For that purpose, the questions were focused on the following factors: user-friendliness, reliability, accuracy, cost, availability, and accessibility. Additionally, the surveyed clinicians were given the freedom to add their opinions on the advantages and disadvantages of PoC devices to find out if other factors influence their readiness to adopt such devices. Demographic data were collected from each person to understand their background and assess their familiarity with the topic without asking for any personal information. Most of the clinicians (81.2%) were between 30 and 49 years old, with an overall age range between 24 and 69 years. A total of 45.8% of the clinicians work in Turkey while the others are in North Syria, but all were Syrian nationals. Given that most of the clinicians were medical doctors, who have considerable practical experience, were well-educated and familiar with PoC devices, this gives the results a high reliability to understand the end user’s perspective regarding the adoption of PoC devices. Moreover, since all clinicians were Syrian, this means that barriers of communication with the patients, who are Syrian refugees, were minimized, which suggests that the results of this work may also reflect the patient’s perspective through discussions and conversations with their healthcare providers.

**Figure 2:**
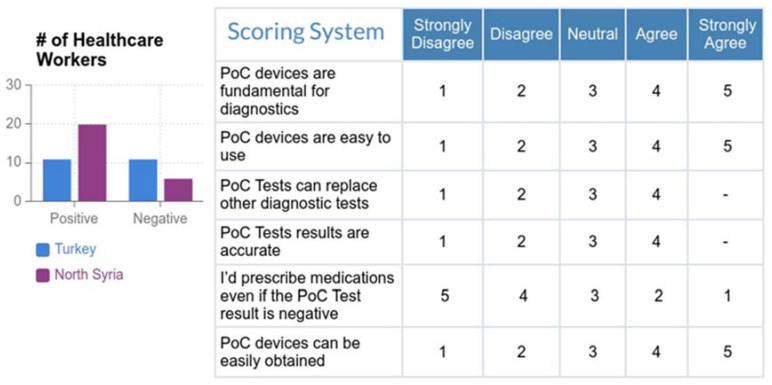
Healthcare workers’ attitudes towards PoC devices. (**Left**) Total number of surveyees was 48 healthcare workers: 22 from Turkey and 26 from North Syria. Surveys are collected anonymously online in both Turkish and Arabic: https://globalchallenges.ku.edu.tr/ (20–31 January 2021). The number of healthcare workers who were positive or negative towards PoC devices is represented based on the country, and shows that healthcare workers in North Syria are more positive compared to those in Turkey. (**Right**) Criteria used to determine the positivity or negativity towards PoC devices and the results based on work location. A total score between 10 and 18 indicates negativity towards PoCs. A total score of 19–28 indicates positivity towards PoCs.

**Figure 3:**
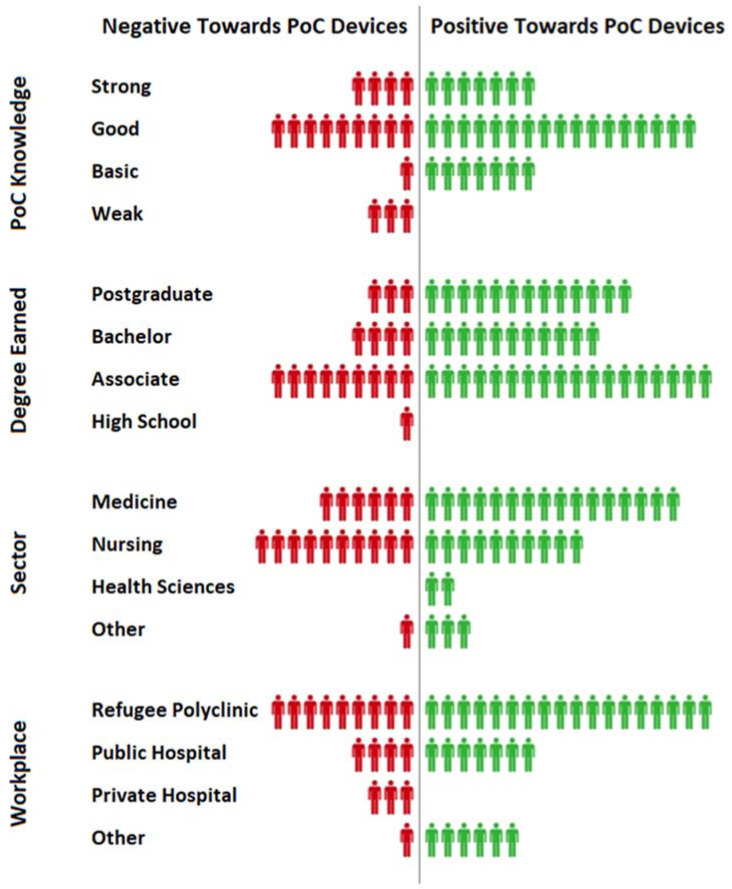
Positivity and negativity of surveyed clinicians towards PoC devices based on their different aspects of experience.

The results of this work showed that healthcare workers were more positive towards PoC devices, especially among those who work in North Syria, where the quality of healthcare was lower than in Turkey. This suggested that PoC devices should be used more frequently in such regions, and they have the potential to be adopted by most of the healthcare workers (Figure 2). To be more specific about the opinion of each demographic group, the workers were categorized based on four factors that reflected their experiences, which were their knowledge on PoC devices, the highest degree earned, the sector they work in, and the workplace. As a result, those who have strong, good, or basic knowledge of PoC devices were more likely to accept and adopt them, unlike those with poor knowledge. This showed that providing basic information about PoC devices was enough to convince the healthcare worker to use them clinically. Workers who had either a postgraduate degree, a bachelor’s degree, or an associate degree all tend to be positive towards PoC devices, while the only worker who was a high school graduate was more negative. In regard to workers’ sectors, all four categories were positive towards PoC devices, and that included medical doctors, nurses, health scientists, and those who worked in other similar sectors. The impact of the workplace was also investigated, and those who work in refugee polyclinics, public hospitals, and other healthcare centers had a positive tendency towards PoC devices, while all three people who worked in private hospitals were negative towards the devices. This could be related to the low price of PoC testing, which was more suitable for public hospitals and polyclinics rather than private hospitals. (Figure 3).

**Figure 4:**
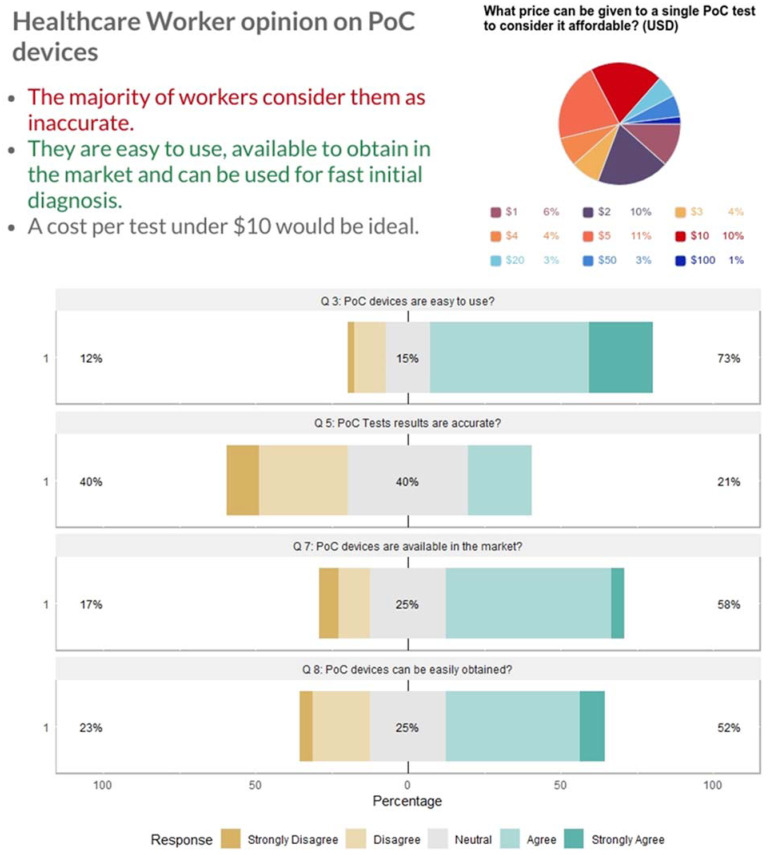
Range of the price of a single PoC test that makes it affordable as seen by the surveyed clinicians (**up**) and degree of agreement with the ease of use accuracy, availability, and accessibility of PoC devices (**down**). Summary statistics of each bar are shown as percentages.

When the opinions of workers were assessed regarding the specifications of PoC devices regardless of their demographic information and experience, there was a general agreement on the importance and advantages of PoC devices. A concern for the majority of workers was the accuracy of PoC test results (Figure 4). These results clearly demonstrated that doctors and healthcare workers preferred to rely on laboratory testing results as a main way of diagnosis and evaluation. Therefore, a test which was accurate rather than more rapid and low-cost was trusted and preferred. The clinicians were also asked to suggest a reasonable price that makes a PoC test affordable, and about 73% suggested a range between USD 1 and 5, 18.75% suggested USD 10 or 20, and 8.33% gave USD 50 or 100 (Figure 4). The last answer may be a result of misunderstanding the question, where the clinicians who answered USD 50 or 100 may have perceived the questions to be asking about a whole device rather than a single assay. However, the answers were not strongly biased to extremely low prices such as USD 1 or 2, suggesting that price was not a big concern, and that can be explained by the fact that Syrian refugees receive free healthcare services in Turkey.

**Figure 5:**
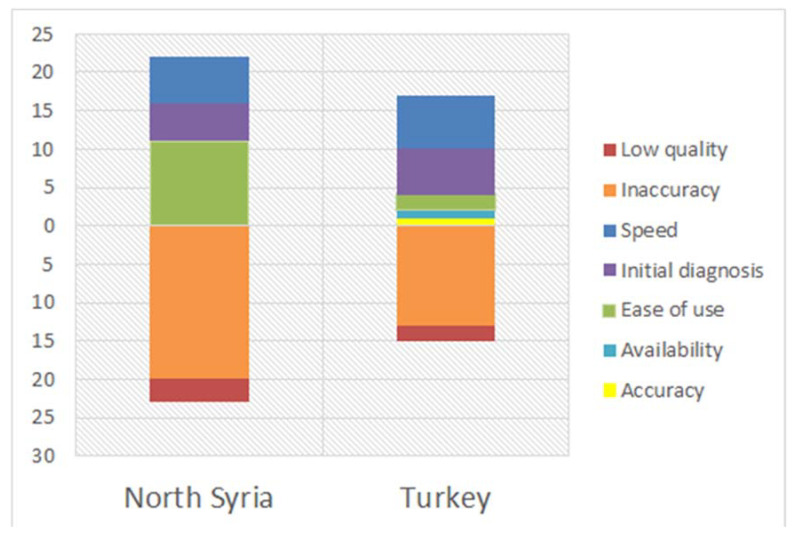
Advantages and disadvantages of PoC devices as seen by the surveyed clinicians based on their work location.

Clinicians were given the chance to write down what they think the advantages and disadvantages of PoC devices would be. The most frequently mentioned advantages were speed and ease of use, which makes sense as those are of the mostly desired features in PoC devices. Only 7.14% mentioned low cost as an advantage, which agreed with the findings of the cost evaluation. An equal small ratio of votes was given to availability, and this also suggests that this factor was not important for the clinicians, or that they do not have accessibility to PoC devices since most of them work in Syria. The least mentioned advantage was accuracy, which consisted only 1.79% of the total number of advantages, agreeing that accuracy is the greatest challenge that faces the adoption of PoC devices among clinicians. Most interestingly, 19.64% of the workers answered that PoC devices were beneficial for initial diagnosis, which was a detail that was not considered in our survey (Figure 5). This suggested advantage shows that, even though most of the clinicians do not trust the accuracy of PoC devices, they might still use them for emergency situations, or to get a quick decision on whether to proceed to laboratory testing or not. In that manner, PoC devices can still be viewed to be desired when considered in that context. Additionally, the newly suggested feature of initial diagnosis can be defined as a combination of other main advantages: speed and ease of use, which are useful in emergency situations. Even though the advantages given were diverse, the disadvantages mentioned by the clinicians were divided into only two categories, the most dominant being inaccuracy with 86.49% of answers. The other option was a clinician-suggested answer, which was low quality (Figure 5). Even though low quality does not constitute a large ratio among the answers, many clinicians mentioned it alongside inaccuracy, which can be viewed as a cause of their low confidence in PoC devices. Specifically, most of the surveyed clinicians were well-experienced, but their experience with PoC devices through the years had caused them to distrust tests due to false negative/positive results. This is informative for PoC device developers and researchers, suggesting that they should focus more on improving the quality of those devices to produce more accurate results, rather than giving more time to enhance the speed, user-friendliness and low cost, which are already established features. By doing this, clinicians can trust PoC devices, resulting in doctors, nurses, and healthcare workers adopting the use of these tests more frequently.

## 5. Conclusions

Point-of-care (PoC) devices can offer an inexpensive alternative for laboratory testing with faster results and fewer instructions to operate, making them easier to use with minimum training [27,28,29,30]. Those advantages allow PoC devices to be suitable for environments with limited resources, such as in refugee camps and warzones [31,32]. An example of this is the recent humanitarian crisis as a result of the Syrian war, which has left millions of Syrian citizens displaced without shelter within neighboring countries such as Turkey or within Northern Syria near the Turkey–Syria border. Due to the large numbers of refugees and the limited conditions of refugee camps, diagnosis and treatment of infectious diseases among refugees are a major challenge. These circumstances cause disease to further spread, increasing infection rate, which impacts the expenditures of healthcare services in neighboring countries and worldwide. Although PoC devices provide an efficient solution to the issue of delayed diagnosis and high healthcare costs, they are still not fully adopted in areas where refugees receive healthcare. In this study, we aimed to understand the obstacles that prevent healthcare workers from using PoC devices to diagnose disease within the Syrian refugee population. For that purpose, we surveyed 48 doctors, nurses, and other healthcare professionals who work with refugees in South Turkey and North Syria, questioning them about their opinions on the most important features of PoC devices, along with their advantages, disadvantages, and whether they would adopt such devices for diagnosis. After analysis of the questionnaire data considering the diverse demographics of the surveyees, we found out that most healthcare workers agree that PoC devices are easy to use, available, and provide fast results. Furthermore, some workers suggested an additional advantage which was that POC devices can be beneficial for initial diagnosis in emergencies. Almost all participants of the survey were in favor of the cost efficiency of such devices. However, the most obvious barrier that appeared to prevent them from adopting PoC devices was their mistrust in the accuracy of their results. To reflect these results within research and industry, developers of PoC devices should dedicate more research and resources to improve the accuracy of results. This will minimize false positive and negative diagnoses. Moreover, target product profiles developed for disease-specific POC tests have to be analyzed to elucidate the link between clinical sensitivity and cost. Since cost effectiveness, user friendliness, and availability are satisfactory enough, in the opinion of healthcare professionals, addressing this issue of accuracy will allow PoC devices to be more widely used for disease diagnosis, especially in areas with large refugee populations.

## Figures and Tables

**Table 1:** Prevalence of risk factors for noncommunicable diseases. High Risk: 3–4 risk factors. Moderate Risk: 1–2 risk factors. Low Risk: 0 factors.

Age (Year)	Non-Communicable Diseases Risks								
	Female			Male			Total		
	Low Risk	Moderate Risk	High Risk	Low Risk	Moderate Risk	High Risk	Low Risk	Moderate Risk	High Risk
18–44	0.2%	53.7%	46.1%	0.3%	45.1%	45.7%	0.3%	49.4%	50.3%
45–69	–	12.9%	87.1%	0.8%	17.5%	81.7%	0.4%	15.1%	84.5%
18–69	0.2%	43.8%	56.1%	0.4%	38.3%	61.3%	0.3%	41.1%	58.7%

## Data Availability

Data is contained within the article.
